# A critical analysis of tinnitus measuring methods

**DOI:** 10.1016/S1808-8694(15)30088-4

**Published:** 2015-10-19

**Authors:** Andréia Aparecida de Azevedo, Patrícia Mello de Oliveira, Adriana Gomes de Siqueira, Ricardo Rodrigues Figueiredo

**Affiliations:** a MD. Otorhinolaryngologist. Partner; b Speech and Hearing Therapist; c Speech and Hearing Therapist; d M.S. in ENT - Federal University of Rio de Janeiro, Assistant ENT Professor - Valença Medical School, RJ. OTOSUL, Otorrinolaringologia Sul-Fluminense

**Keywords:** scales, measurement, tinnitus

## Abstract

One of the main factors that make tinnitus treatment so difficult is the subjectivity of measuring methods and therapeutic monitoring. **Database:** Our aim, in this study, is to make a critical analysis of tinnitus measuring methods. **Conclusion:** There is no consensus about tinnitus measuring methods, causing criticism in the methodology used in many papers. In Brazil, the simplest methods are the most used.

## INTRODUCTION

Tinnitus may be defined as a hearing sensation that does not come from outside, seeming to come from one or both ears, or even from the head, without a precise source location^1^. It is, in fact, a symptom, and not a disease, and according to varied studies, it affects between 14 and 32% of the population, and it may be disabling in up to 5% of the cases2. It may even be a sequela of different pathological processes which are no longer active^2^.

Among the many classifications proposed for tinnitus, the one most accepted today classifies tinnitus into Auditory (caused by alterations in the ear, auditory pathways and auditory cortex) and Para-auditory (caused by vascular and muscle structures near the ear and auditory pathways) ^2^.

Tinnitus physiopathology has yet many gaps of knowledge to be filled, and it is believed that in many cases it is multifactorial^2^. Among the major theories proposed, we list excitotoxicity^2^, ^3^, ^4^, lesions in the efferent system^2^, ^5^, tectorial membrane collapse^6^, correlation with pain^7^ and the activation of psychosomatic and autonomic loops^8^. It is possible that, in the future, a theoretical model becomes part of these theories.

Besides the difficulties brought about by the lack of understanding in physiopathology, there is also some difficulty in measuring tinnitus and, consequently, in assessing therapeutic results^9^. We still lack a consensus on the ideal methodology to measure tinnitus. It may be extremely subjective and influenced by environmental and psychosomatic factors. Many treatment results with medication and other means of treatment are disputed by the tinnitus measuring criteria employed^10^, ^11^.

In the present paper we intend to carry out a critical analysis of the main methods used today to measure tinnitus.

### Acuphenometry

It means “to measure acuphens” (tinnitus). This is one of the oldest methods and one of the least employed today. It encompasses a set of audiologic techniques used to try find in tonal audiometry a pure tone similar to the patient’s tinnitus^6^.

According to some authors, it could also be useful in the topo-diagnosis of dysacousia, starting from the idea that conductive dysacousia would generate tinnitus of lower frequencies (“water fall”, “ocean waves”); and sensorineural dysacousia would generate higher frequency tinnitus (“cicada”, “whistle”, “cricket”). Mixed dysacousia would have a varied behavior, generating low or high tinnitus^2^, ^6^.

Acuphenometry is highly dependent on the patient’s intellectual capacity and concentration, including certain skills to perceive sounds of different frequencies. It may work exceptionally well with musicians. It is also greatly examiner-dependent. In cases of unilateral tinnitus, the test is easier, since it compares the tinnitus with the sounds made to the contralateral ear. In bilateral cases, the attempts are gradual and successive, giving the ear higher and lower tones until it comes to one sound that more closely resembles the patient’s tinnitus. Sound masking is necessary in most of the cases6. Most studies classify most of the tinnitus in the higher frequencies, notably between 6 and 8 kHz^6^.

Acuphenometry has the major advantage of allowing for tinnitus monitoring, thus, treatment monitoring; and it also aids in the topo-diagnosis of auditory lesions. However, there are some disadvantages. First, tonal audiometry tries to correlate pure tones and tinnitus, which in many cases is multitonal. Moreover, few patients are able to provide an accurate correlation of their tinnitus with pure tones, even with experienced examiners. Acuphenometry is highly important in the treatment by masking, which is today increasingly less used. In our opinion, for acuphenometry, the cons outweigh the pros in this method.

### Analogue and Visual Scale (AVS)

This is the scale that has been more frequently used in Brazilian tinnitus studies^10,12-14^. Actually, it is a visual graph used to determine the level of disturbance or discomfort caused by tinnitus, in a scale from 1 to 10. Intensity and discomfort are the most valued items.

**Figure 1:**
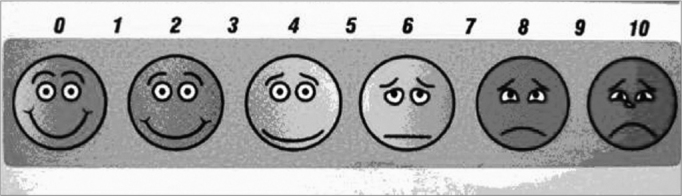
Analogue-Visual Scale model (AVS).

As the examiner asks the patient to determine in a 1 to 10 scale, how much the tinnitus bothers him/her, without a visual aid (as shown in figure 1), there may be score variations that the patient may attribute to different things, according to the variables related to psychosomatic and intellectual factors. The use of an image produces a visual reinforcement that may be remembered by the patient him/herself.

AVS’s main advantage, besides the visual reinforcement and response standardization, is its very simplicity; such factor may be decisive in intellectually challenged patients. Nonetheless, this same simplicity may be seen as a disadvantage of this method, since it may induce a superficial and variable problem in assessment, and so, ideally, it must be associated with other methods. We believe that for the Brazilian population, this is still the most applicable assessment method.

### Tinnitus Handicap Inventory (THI)

Increasingly, methods that measure not only the direct effects of a disease, but also the effects such disease has on the patients’ lives have been used^15^, ^16^. In 1996, Newman, Jacobson and Spitzer published an article about the development of the Tinnitus Handicap Inventory, through the observation and criticism of other methods such as the Tinnitus Handicap/Support Questionnaire, Tinnitus Effect Questionnaire, Tinnitus Severity Questionnaire and Tinnitus Reaction Questionnaire^15^. According to the authors, their main goal was to create a method with the following characteristics:

• Summarized, adequate to daily clinical practice;
• easy deployment and interpretation;
• approaching different tinnitus impacts on the patient’s life quality;
• validity and reliability.

Clinical data from tinnitus patients and data from other scales were used in developing the THI. Three major items are assessed by the THI, and they are^15^:

• tinnitus-related function reactions, such as difficulty to concentrate and anti-social behavior;
• tinnitus-related emotional reactions, such as anger, frustration, irritability and depression;
• tinnitus-related catastrophic reactions, such as despair, hopelessness, fear of “severe disease”, loss of control and incapacity to cooperate.

**Chart 1:**
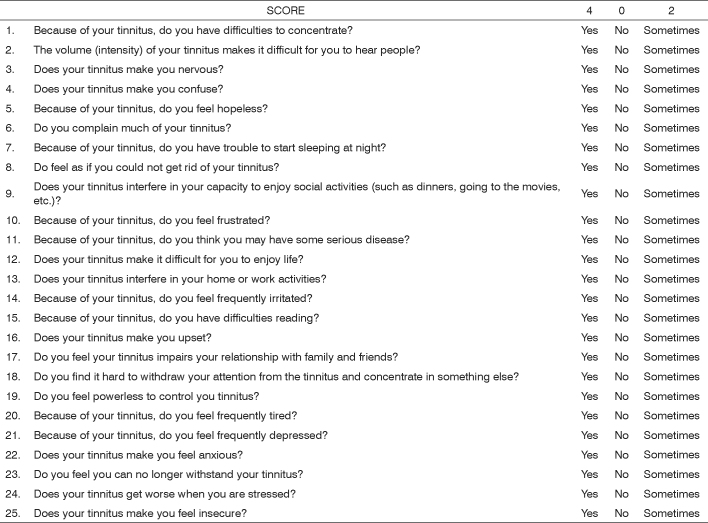
Tinnitus Handicap Inventory questionnaire, copied from Ferreira Pea et al.^17^

**Chart 2:**
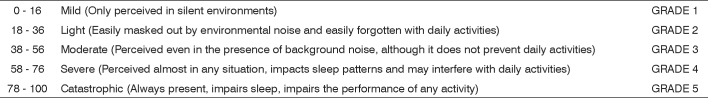
Tinnitus Handicap Inventory scores and interpretation, from Newman et al.

Below are the original THI questionnaire (Chart 1), and its interpretation (Chart 2):

In our view, in relation to the authors’ initial proposal, the THI is more complex than the AVS; however still usable in the daily clinical practice. For the Brazilian Population, it may not be of easy application in many situations. As to the tinnitus aspect items used, its validity and reliability leads us to believe it is a valid method is more complex than AVS; however still applicable in routine clinical practice. For the Brazilian population, it may be hard to apply in many situations. As to the items used to approach the numerous aspects related to tinnitus, validity and reliability, we believe it is the most valid method.

### Hospital Anxiety and Depression Scale (HADS)

According to the authors, this scale can be summarized and is of easy use. Its main goal is to separate the psychological aspects from those purely somatic. HADS is made up of 14 items (each with scales from 0 to 3), each subdivided in 11 subscales of 11 items each, assessing aspects related to anxiety and depression^18^.

In our opinion, this scale, besides being relatively complex, evaluates only the tinnitus psychosomatic aspects, thus being incomplete.

### Tinnitus Disturbance Simplified Scale Analysis (TDSSA)

Seeking a simplified method to quantify tinnitus, prioritizing aspects related to the patient’s daily life that is affected by the tinnitus, we developed this scale in 2004.

Our proposal considers individual characteristics and occasional psychological alterations found in patients with tinnitus^19^. It stems from the clinical observation of outpatient ward patients and those patients who participated in the 1st PAZ (Tinnitus Self-Help Program), held in Volta Redonda, in 2002^4^. In this program, we held talks by otorhinolaryngologists, hearing therapists and physical therapists, also patient integration with reports from their individual experiences regarding their tinnitus, based on other similar groups, including the GAPZ (Tinnitus Patients’ Support Group), pioneer in Brazil, at the Federal University of São Paulo^2^.

The scale classifies the degree of discomfort caused by tinnitus from 0 to 10, according to the following characteristics^19^:

(1) Tinnitus perceivable only in silent environments, not interfering with sleep patterns;
(2) Tinnitus perceivable only in silent environments, makes the patient, at least occasionally, to have trouble falling asleep, does not lead the patient to seek medical help in order to get prescriptions for sleeping pills;
(3) The same situation above mentioned, but here the patient seeks medical help to get prescriptions for sleep inducing medication;
(4) Tinnitus perceivable during the day, easily masked by environmental noise;
(5) Tinnitus perceivable during the day makes the patient avoid silent environments;
(6) Tinnitus perceivable during the day, causing emotional instability/irritability;
(7) Tinnitus interferes with professional, schooling and/or domestic activities, reducing performance in general;
(8) Tinnitus causing reclusion and anti-social behavior (isolation, aggressiveness, etc.);
(9) Tinnitus causing hospital admittance for psychiatric reasons;
(10) Tinnitus makes the patient consider drastic actions, such as inflicting deafness, cutting the ear off and suicide.

In type 2 tinnitus, the symptom draws the patient’s attention, however, often times he/she does not seek medical help. Starting on type 3, patients usually seek medical care. From type 5 on, associated psychological alterations become increasingly more important. In the extreme case of type 10, we may mention the cases of patients that would have committed suicide because of tinnitus (there are cases reported in both, the national and international literature), and patients who ask the physician to destroy their auditory nerve^19^.

Although it may be argued that this scale does not assess the tinnitus symptom in an objective way, since if we could objectively measure tinnitus we would have obtained different scores in this scale for patients with tinnitus of the same intensity, its major value lies in assessing symptom progression and the therapeutic effects in one given patient, since it is based on a patient’s daily routine^19^.

We believe the main criticism to this scale lies in its very simplicity that may suggest a superficial tinnitus assessment. Moreover, by including items related to sleep and psychiatric disorder treatments must be analyzed carefully. As positive factors, we mention the ease of application by the physician, who may quantify tinnitus through questions belonging to his own anamnesis (the patient does not have to answer a specific questionnaire, since this may inhibit some patients), and it also values the items that most disturb the patient in his/her daily activities.

### Animal models for tinnitus

For many years an animal model was searched, aiming at analyzing the behavior of an animal supposedly with tinnitus, and it would also allow the study of drugs in lab animals. The major problem was how to be sure the animal had tinnitus^9^, ^20^.

The first attempts to establish an animal model for tinnitus started in the late 70’s, through techniques that measured the 2-deoxiglucose (2-DG) amount in the animal’s brain. It is known that the only source of energy used by neurons is glucose. 2-DG is a glucose analogue, which is partially metabolized, and the rest is built up in the physiologically active tissue. It was then proposed that its building up was an indirect indication of an increase in brain metabolism^9^, ^21^.

Lab animals suffered unilateral destruction of the cochlea and auditory nerve and were analyzed as to the building up of 2-DG, at 2, 7 and 21 days after the procedure. It was then observed that the 2-DG concentration suffered a reduction early on, and later reached levels that were similar to those found in the intact side. It was then proposed that this supposed recovery of metabolic activity happened because of tinnitus development in these animals, since we know that in humans, about 50% of the patients submitted to unilateral cochlea and auditory nerve destruction develop tinnitus^9^, ^21^.

The problems that culminated with the little acceptance of this model were the doubts related to the correlation between metabolic activity recovery and the presence of tinnitus, and the very fact that in humans only 50% of the patients who undergo cochlear destruction develop tinnitus^9^.

Then, the conditioned behavioral models became the goal to be reached^9^, ^20^, ^22^. The models developed during the 80’s and 90’s by Jastreboff et al. are currently the most accepted by the international scientific community. We will try, in a simplified way, to expose the very basis of these models.

The basic idea is to create associations between silence and danger and, inversely, between noise and safety. Thus, correlating tinnitus with the perception of noise, we expect that the animal with tinnitus would not have fear reactions in a silent environment^9^, ^20^.

Initially, the animals (i.e. rats) underwent partial water deprivation, monitored by animal weight control. In a separate chamber, tubes supplied water to the animals and the water intake was measured. Later on, the animals were conditioned to fear silent environments, since they received electrical discharges whenever the environmental noise was suppressed. Consequently, in the silent water tube filled chamber there was a reduction in the number of times these animals sought water. Fear associated with silence may be later on abolished with repetitive suppression of environmental noise no longer associated with electrical discharges^9^, ^20^.

Then they compared the necessary conditioning time for both, the fear and silence association and the dissociation, in rats to which salicylates were given (which produce, depending on the dose and length of time using it, tinnitus in almost 100% of human beings), with control groups that received only saline solution. Statistically significant differences between these two groups allowed the conclusion that the salicylate exposed rats were effectively with tinnitus. The animals that receive salicylate took longer than those in the control group to make and to unmake the association between silence and fear using electrical discharges and later suppressing them. This happened because they had tinnitus20. In another experiment, the introduction of salicylate was compared to the introduction of sounds (that mimicked tinnitus), with similar results in both groups^9^.

It was proven that salicylates have their action restricted to the auditory system of rats, and tests with other ototoxic drugs, such as quinine, presented similar results, strengthening the model. Moreover, these models allow for frequency (through the exposure to different sounds during fear suppression) and intensity (through the observation, in humans, that the salicylate toxicity is proportional to the dose used) evaluation. Later studies with these models have corroborated with the understanding of the association between tinnitus and the calcium homeostasis, with the anomalous activity of the auditory pathways and with the Tinnitus Retraining Therapy (TRT)^9^ itself.

Other models were developed, based on the same conditioning principle. Animal models are useful in order to understand the tinnitus physiopathology and also its treatment, prior to tests with human beings. Notwithstanding, they require a complex and expensive laboratorial structure. Unfortunately, the animal models do not solve the problem of not having an objective model of tinnitus measurement in humans. Such model would represent a watershed in understanding and treating tinnitus. Meanwhile, our only alternatives for clinical tests in humans are the scales and questionnaires.

## CONCLUSION

We still lack consensus as to tinnitus measuring methods and, as a consequence, the assessment of new therapeutic approaches is always questioned in its methodology. In reality, in the Brazilian Health Care System, the simplest scales, such as the AVS, are the easiest applicable, thus being the most employed. THI represents the most complete evaluation questionnaire, however of difficult application. The TDSSA, even with its limitations, may represent an intermediate alternative. Animal models, of difficult execution, represent the ideal pre-clinical models. Acuphenometry and the HADS, because of their large limitations, today are very little used in assessing tinnitus.

## References

[1] Meyer B, Meyer B (2001). Acouphènes et Hyperacousie.

[2] Azevedo AA, Figueiredo RR (2004). Atualização em Zumbido. Rev Bras Otorrinolaringol, Caderno de Debates.

[3] Bonfils P, Puel JL, Meyer B (2001). Acouphènes et hyperacousie.

[4] Cunha N, Puel JL. Zumbidos, presente e futuro: da investigação à aplicação clínica. In: http://www.otoneuro.pt/fórum; 2000

[5] Fávero M. (2003). Estudo das vias auditivas eferentes em indivíduos com zumbido [Doutorado].

[6] Menezes P, Santos Filha VAV (2005). Acufenometria: o resgate de um instrumento de avaliação do zumbido e sua correlação com perdas auditivas sensoriais. Revista Fonoaudiologia Brasil.

[7] Boureau F, Meyer B, Meyer B (2001). Acouphènes et hyperacusie.

[8] Hazell JWP, Jastreboff PJ (1990). Tinnitus Internal Auditory mechanisms: a model for tinnitus and hearing impairment. J Otolaryngol.

[9] Jastreboff P, Sasaki CT (1994). An animal model of tinnitus: a decade of development. Am J Otol.

[10] Azevedo A, Figueiredo RR (2005). Uso do acamprosato no tratamento do zumbido: um estudo duplo-cego. Rev Bras Otorrinolaringol.

[11] Crítica ao estudo “Tinnitus treatment with acamprosate: a double-blind study”, http://www.newswise.com/articles/view/514635, Setembro de 2005 10.1016/S1808-8694(15)31266-0 PMC9441991 16612523

[15] Newman C, Jacobson GP, Spitzer JB (1996). Development of the Tinnitus Handicap Inventory. Arch Otolaryngol- Head Neck Surg.

[16] Winfried R, Weise ND, Kley ND, Martin A (2005). Psychophysiologic Treatment of Chronic Tinnitus: A Randomized Clinical Trial. Psychosomatic Medicine.

[17] Ferreira PEA, Cunha F, Onishi ET, Branco FCA, Ganança FF (2005). Tinnitus Handicap Inventory: adaptação cultural para o português brasileiro. Pró-fono.

[18] Andersson G, Kaldo-Sandström V, Ström L, Strömgren T. (2003). Internet administration of the Hospital Anxiety and Depression Scale in a sample of tinnitus patients. J Psychosomatic research.

[19] Figueiredo RR, Azevedo AA (2004). Incômodo gerado pelo zumbido: uma proposta de classificação.

[20] Jastreboff P, Brennan JF, Coleman JK, Sasaki CT (1988). Phantom Auditory Sensation in Rats: An Animal Model for Tinnitus. Behavioral Neuroscience.

[21] Sasaki C, Kauer JS, Babitz L (1980). Differential [14C]2-deoxyglucose uptake after deafferentation of the mammalian auditory pathway-a model for examining tinnitus. Brain Research.

[22] Sasaki C, Kauer JS, Babitz L (1981). Tinnitus: development of a neurophysiologic correlate. Laryngoscope.

